# An Olive-Derived Extract 20% Rich in Hydroxytyrosol Prevents β-Amyloid Aggregation and Oxidative Stress, Two Features of Alzheimer Disease, via SKN-1/NRF2 and HSP-16.2 in *Caenorhabditis elegans*

**DOI:** 10.3390/antiox11040629

**Published:** 2022-03-25

**Authors:** Jose M. Romero-Márquez, María D. Navarro-Hortal, Victoria Jiménez-Trigo, Pedro Muñoz-Ollero, Tamara Y. Forbes-Hernández, Adelaida Esteban-Muñoz, Francesca Giampieri, Irene Delgado Noya, Pedro Bullón, Laura Vera-Ramírez, Maurizio Battino, Cristina Sánchez-González, José L. Quiles

**Affiliations:** 1 Department of Physiology, Institute of Nutrition and Food Technology “José Mataix Verdú”, Biomedical Research Centre, University of Granada, 18100 Armilla, Spain; romeromarquez@ugr.es (J.M.R.-M.); mdnavarro@ugr.es (M.D.N.-H.); victoriajt@correo.ugr.es (V.J.-T.); pedrollero@correo.ugr.es (P.M.-O.); tforbes@ugr.es (T.Y.F.-H.); laura.vera@genyo.es (L.V.-R.); 2 Department of Nutrition and Bromatology, University of Granada, 18071 Armilla, Spain; aidaem@ugr.es; 3 Department of Clinical Sciences, Polytechnic University of Marche, 60131 Ancona, Italy; f.giampieri@univpm.it (F.G.); m.a.battino@staff.univpm.it (M.B.); 4 Department of Biochemistry, Faculty of Sciences, King Abdulaziz University, Jeddah 21589, Saudi Arabia; 5 Research Group on Foods, Nutritional Biochemistry and Health, Universidad Europea del Atlántico, Isabel Torres, 21, 39011 Santander, Spain; irene.delgado@uneatlantico.es; 6 Department of Periodontology, Dental School, University of Seville, C/Avicena, s/n, 41009 Seville, Spain; pbullon@us.es; 7 Department of Genomic Medicine, GENYO: Centre for Genomics and Oncology (Pfizer-University of Granada and Andalusian Regional Government), PTS Granada, 18016 Armilla, Spain; 8 International Joint Research Laboratory of Intelligent Agriculture and Agri-products Processing, Jiangsu University, Zhenjiang 212013, China; 9 Sport and Health Research Centre, University of Granada, C/Menéndez Pelayo 32, 18016 Armilla, Spain

**Keywords:** age-related diseases, antioxidants, HSP-16.2, neuroprotection, *Olea europaea*, olive by-products, polyphenols, RNAi, tau protein

## Abstract

Olive milling produces olive oil and different by-products, all of them very rich in different bioactive compounds like the phenolic alcohol hydroxytyrosol. The aim of the present study was to investigate the effects of an olive fruit extract 20% rich in hydroxytyrosol on the molecular mechanisms associated with Alzheimer disease features like Aβ- and tau- induced toxicity, as well as on oxidative stress in *Caenorhabditis elegans*. Moreover, characterization of the extracts, regarding the profile and content of phenolics, as well as total antioxidant ability, was investigated. The study of lethality, growth, pharyngeal pumping, and longevity in vivo demonstrated the lack of toxicity of the extract. One hundred μg/mL of extract treatment revealed prevention of oxidative stress and a delay in Aβ-induced paralysis related with a lower presence of Aβ aggregates. Indeed, the extract showed the ability to avoid a certain degree of proteotoxicity associated with aggregation of the tau protein. According to RNAi tests, *SKN-1/NRF2* transcription factor and the overexpression of *HSP-16.2* were mechanistically associated in the observed effects.

## 1. Introduction

The extraction of olive oil from the fruit of the olive tree (*Olea europaea* L.) leads to a large amount of by-products very rich in bioactive compounds [1]. Many of these compounds have been recognized to present antioxidant properties and to have different effects on cell biology, which has attracted the interest of pharmaceutical and functional food industries [2]. Furthermore, re-use of the waste products could reduce the environmentally negative impact of the olive grove sector, contributing, at the same time, to a circular economy [2]. Hydroxytyrosol is a phenolic compound with extremely high antioxidant activity which constitutes one of the main components of the minor fraction of extra virgin olive oil. This compound comes from oleuropein hydrolysis, and it is present in the fruit and also in the olive leaves. After olive oil production, a large concentration of hydroxytyrosol remains in the waste fraction [3]. Many health benefits have been attributed to hydroxytyrosol, including improvement of serum lipid profile [4], cardioprotection [5,6,7,8], and anti-diabetic [4], anti-neoplasic [9,10] and anti-inflammatory properties [4]. Moreover, effects against neurodegenerative diseases have been reported [11,12]. Regarding neurodegeneration, Alzheimer Disease (AD) is the most relevant type of dementia, having high social, physical, physiological and economic impact [13]. AD is an age-related disease and, since the aged population is continuously growing, together with the absence of effective pharmacological treatments, there is continuous interest in investigating new strategies to attenuate or retard the onset of AD, or to provide treatments for this dementia. Indeed, it has been recognized that dietary antioxidants and phenolic compounds might provide neuroprotection regarding AD [14,15]. Accordingly, the development of new functional foods and nutraceuticals useful to prevent or treat AD, based on the healthy properties of hydroxytyrosol and some olive by-products, would be of great interest.

The experimental model *Caenorhabditis elegans* has been widely used to investigate, among others, aspects related to oxidative stress, aging, neurodegenerative diseases, and longevity, and in studies regarding biomedical properties of food-derived compounds. One aspect to point out concerning this experimental model is the existence of RNAi libraries and transgenic strains with genes coupled with GFP, which makes this worm very versatile, offering more sensitive and efficient analyses. Considering all the above-mentioned, the objective of the present research was to study, focusing on some very relevant features of AD, a 20% hydroxytyrosol-rich olive fruit extract (HOFE) already authorized for human nutrition. Firstly, characterization of the extract concerning antioxidant properties, total phenolic and flavonoid content, and the profile of individual phenolic compounds, were analyzed. Then, the in vivo experimental model *C*. *elegans* was employed for the evaluation of different aspects associated with any toxicity associated with the extract and to evaluate the potential benefits regarding three well-known features of AD: oxidative stress, and Aβ and Tau proteotoxicity. Molecular mechanisms behind observed effects were investigated by using RNAi technology and GFP-coupled transgenic strains.

## 2. Materials and Methods

### 2.1. Reagents and Chemical Products

All chemicals were procured from Panreac Química (Barcelona, Spain), Thermo Fisher (Waltham, MA, USA), Labbox labware (Barcelona, Spain), Merck (Darmstadt, Germany) or Roche (Basel, Switzerland). Reagents and solvents were of the highest available purity. A Millipore (Milford, MA, USA) purification system was used to obtain double distilled deionized water.

### 2.2. Extract Preparation

*Olea europaea* fruit dry extract (DE) 20% rich in hydroxytyrosol was generously donated by Natac (Madrid, Spain). Hydroxytyrosol-rich Olive Fruit Extract (HOFE) was obtained from crushing and mixing olives to get the juice, which was then purified and concentrated. One hundred μg/mL of the dry extract diluted in ethanol/water (25:25, *v*/*v*) was used for most of the assayed tests.

### 2.3. Analysis of Total Phenol and Total Flavonoid Amount and Total Antioxidant Capacity

To determine total phenol content the Folin-Ciocalteu method was performed [16]. Briefly, samples were mixed with Folin-Ciocalteu reagent for 5 min. Na_2_CO_3_ was added and the mixture placed at room temperature in the dark for 2 h. Seven hundred and sixty nanometers of absorbance were measured. As standard, a dilution of a known concentration of gallic acid was used. Results are presented as milligrams of gallic acid equivalent/gram of dry extract. Total flavonoids content was assayed by reacting samples with NaNO_2_ and AlCl_3_ for 6 and 5 min., respectively. After that, the addition of NaOH was performed followed by reading absorbance at 510 nanometers [17]. Catechin was employed as standard, expressing results as milligrams of catechin equivalent/gram of dry extract. Total antioxidant power of HOFE was measured by FRAP, DPPH and ABTS methodologies. FRAP evaluates the samples’ ability to reduce from Fe^3+^ to Fe^2+^. The reaction is carried out in an acid medium with sodium acetate solution. The color changes when Fe, forming a complex with 2,4,6-tripyridyl-*s*-triazine (TPTZ), is reduced. Reduction level is tested by registering absorbance at 593 nm [18]. DPPH analysis was performed following the Kumaran and Karunakaran procedure [19]. In this test, the color of the free radical is eliminated by the antioxidants present in the sample when absorbance is determined at 517 nm. The ABTS method is based on reduction of the free radical on account of the sample’s antioxidants. Previously, a radical was produced by oxidizing ABTS with K_2_S_2_O_8_. The absorbance values were registered at 734 nm and presented in terms of milligrams of Trolox equivalent/gram of dry extract [20]. All analyses in this section were performed in a minimum of three replicates by using the microplate reader Synergy Neo2 (Biotek, Winooski, VT, USA).

### 2.4. Identification of Individual Phenolic Compounds

To identify hydroxytyrosol in HOFE, a method based on UPLC-QTOF-MS/MS was employed by using conditions previously described [21]. MassLynx V4 software (Waters Laboratory Informatics, Mildford, MA, USA) was used to compare the obtained molecular ions and fragments with data present in scientific literature regarding research conducted on olive leaf extracts.

### 2.5. Caenorhabditis elegans Usage

The following strains of *C*. *elegans* were employed in the present research: N2 Bristol (wild type), CL802 (smg-1(cc546) I; rol-6(su1006) II), CL4176 (dvIs27 [myo-3p::A-Beta (1–42)::let-851 3′UTR) + rol-6(su1006)] X), TJ356 (zIs356[daf-16p::daf-16a/b::GFP + rol-6(su1006)]), LD1 (ldIs7 [skn-1b/c::GFP + rol-6(su1006)]), CL2166 (dvIs19 [(pAF15)gst-4p::GFP::NLS] III), BR5706 (byIs193 [rab-3p::F3(delta)K280 + myo-2p::mCherry], TJ375 (gpIs1[hsp-16.2::GFP]) and CF1553 (mu1s84[pAD76(sod-3::GFP) + rol-6(su1006)]), bkIs10 [aex-3p::hTau V337M + myo-2p::GFP]). Strains were maintained at 20 °C in a VELP 120E incubator (Usmate, Italy). CL802 and CL4176 were cultivated at 16 °C. The nematodes were grown in a standard NGM medium with a lawn of OP50 *E*. *coli* as food. All strains come from the Caenorhabditis Genetics Center (Minneapolis, MI, USA). To start all experiments, worms were synchronized by bleaching.

### 2.6. Toxicity Assays

Toxicity of the extract was tested by different procedures, including 24 h lethality, assessing of pharyngeal pumping, the growth test, analysis of fertility and reproduction, as well as by the measurement of intracellular ROS content and by lifespan analysis. Concerning 24 h lethality, the wild strain N2 was used to assess the acute toxicity of the extract in *C*. *elegans*. L4 age-synchronized nematodes were moved to 24 well plates which contained rising concentrations of the treatment (0, 0.1, 1, 10, 100, 1000 μg/mL) without a food source. After 24 h of incubation at 20 °C, a dissecting microscope (Motic Inc., Ltd., Hong Kong, China) was used to score the live or dead animals. Dead status was attributed when animals were not able to respond to mechanical stimulus. For each concentration, three independent assays were performed, each one with at least three NGM plates containing at least five nematodes per dish. Results were presented in percentage terms of 24 h survival. Most of the following experiments were developed by using the 100 μg/mL dosage. Pharyngeal pumping score was used to evaluate the influence of the treatment on worm metabolism. N2 synchronized eggs were routinely grown in dishes containing or not containing 100 μg/mL of the extract. The plates were maintained for four days at 20 °C. After that time, worms were moved onto normal fresh dishes with food for scoring pharynx bulb contraction per minute, using a similar microscope as the one used for lethality. Three independent experiments, with at least ten nematodes each, were performed. Results are presented in terms of number of pharyngeal pumps/min. To analyze the effect of the HOFE on worm development, a growth test was performed. The worms and the plates were prepared as in the above-described experiments. Pictures from worms aged 4 days, belonging to all experimental groups, were captured with the same microscope as that mentioned above. Nematode size (μm) was analyzed by using Motic Images Plus 3.0 (Motic Inc., Ltd. Hong Kong, China) software. As for previous experiments, three replicates were assayed, having a minimum of 20 worms each. For the analysis of fertility and reproduction, L4 stage N2 synchronized nematodes were placed in control or treated (100 μg/mL) plates containing OP50 *Escherichia coli* for 24 h at 20 °C. After exposition, nematodes were individually moved to a dish containing OP50 bacteria without experimental treatment. Every day, until the end of the egg laying phase, animals were transferred to fresh plates without treatment. For each worm, eggs were counted daily for each nematode and the eggs hatching into larvae were counted the day after using the same dissection microscope as above. The experiment was repeated in triplicate with a minimum of 10 nematodes per treatment. Fertility was represented as total number of larvae, and reproduction was shown as total number of eggs.

ROS content was measured in the worms through the 2′,7′-Dichlorofluorescein Diacetate (DCFDA) technique [22]. N2 synchronized eggs were transferred on plates containing or not containing 100 μg/mL of HOFE for 48 h at 20 °C. Then, control and treated nematodes were washed and exposed, for 15 min, to 2.5 mM of 2,2′-Azobis(2-methylpropionamidine) dihydrochloride (AAPH) to induce oxidative stress. An unexposed control group in basal conditions was added and treated with the vehicle (water) to verify the oxidative stress induction was effective. After that, M9 was used to remove the inductor followed by incubation under 25 μM DCFDA for 2 h at 20 °C. The Union Biometrica Biosorter cytometer (Holliston, MA, USA) was used to measure the fluorescence intensity of at least 200 individuals per experimental group. Results are presented as arbitrary units of fluorescence intensity.

Long-term toxicity was assessed by the lifespan study. Eggs of N2 strain were transferred into plates with 0 or 100 μg/mL of HOFE and bacteria lawn and maintained at 20 °C. The first day of the experiment was considered to be when nematodes reached adulthood. This day, 120 animals were placed into fresh dishes, again with or without the treatment and the OP50 as source of food. That transference and the live worm count were performed every day. Dead status was attributed when animals did not express any movement after repeated mechanical stimulation. Nematodes were considered as censored when they were lost out of the dish or after extrusion of internal organs. A Kaplan-Meier curve was used to present lifespan study.

### 2.7. Paralysis Evaluation and Amyloid-β Aggregate Staining

Evaluation of worm paralysis was developed by using CL802 and CL4176 strains. CL4176 are transgenic worms sensitive to temperature which produces β1–42 human amyloid peptide in the cells of the muscle wall, whereas CL802 is a non-paralyzable control strain. Synchronized eggs were collocated in plates with or without 100 μg/mL of the treatment and seeded with OP *Escherichia coli*. Animals were grown at 16 °C. After 48 h, the temperature in the worm incubator was raised to 25 °C to induce β1–42 peptide expression. Paralysis level was registered from 20 h with temperature being increased every two h up to 32 h. The absence of response to mechanical stimulus was considered to indicate paralysis. Experiments, performed in triplicate, were performed with 20 worms per group each.

Thioflavin T stain was carried out to identify Aβ aggregation. CL4176 worms were treated as in the paralysis assay. After 26 h of temperature shifting, the animals washed with M9 were fixed for 24 h with 4% paraformaldehyde (pH 7.4) at 4 °C. Then, worms were permeabilized by using a buffer at 37 °C made of 5% β-mercaptoethanol, 125 mM Tris and 1% Triton X-100, pH 7.4. After 24 h, to remove the buffer, two washes with M9 were performed. Then, worms were stained with a solution of 0.125% Thioflavin T in 50% ethanol. After 30 min, samples were faded with sequential ethanol washes. Images of the stained animals were taken with a Nikon epi-fluorescence microscope (Tokyo, Japan) at 40×. CL4176 untreated worms were considered to be the positive control, while non-treated CL802 nematodes were considered the negative control.

### 2.8. Locomotion Analysis

The transgenic strain BR5706 was used for evaluating the HOFE effect on another feature of AD physiopathology. These nematodes present a pan-neuronal and constitutive expression of the human Tau pro-aggregating protein. This defect results in adult nematodes with defects in locomotion. Age-synchronized eggs from BR5706 were incubated on NGM dishes with 0 or 100 μg/mL HOFE at 20 °C for 72 h. After that time, the nematodes were placed on slides with 20 μL M9. Then, the WormLab Imaging system (MBF Bioscience, Williston, VT, USA) was used to record, track, and analyze movement. As function markers of locomotive behavior, wavelength, swimming speed and wavelength dynamic amplitude were used in experiments that were performed in triplicate.

### 2.9. Transgenic Reporter Assay for DAF-16/FOXO, SKN-1/NRF2, SOD-3, HSP16.2 and GST-4

Transgenic strains TJ356, LD1, CF1553, TJ375 and CL2166 were used with the aim of delving into the effect of HOFE on molecular pathways and the mechanisms underlying previously observed effects. The TJ356 strain contains the DAF-16::GFP fusion protein. LD1 worms express SKN-1 transcription factor fused with GFP in the intestine and ASI neurons. CF1553 expresses SOD-3 fused with GFP as well. HSP-16.2 and GST-4 are contained in the strains TJ375 and CL2166, respectively.

For all the gene-reporter strains, worms were grown from eggs in plates with or without HOFE 100 μg/mL. After 48 h, animals were placed on glass slides with sodium azide to decrease worm mobility. A Nikon epi-fluorescence microscope (Eclipse Ni, Nikon, Tokyo, Japan) was fitted with a Nikon DS-Ri2 camera (Tokyo, Japan) and used for the acquisition of worm images at 10× magnification for all the strains, except for TJ375, which was captured at 40× magnification. NIS-Elements BR (Nikon, Tokyo, Japan) software was used for image analysis. For the analysis of TJ356, three patterns were considered, and a semi-quantitative scale was applied: cytosolic location was represented by the value ‘1’, intermediate by ‘2’ and nucleation adopted the number 3 [22]. In LD1 worms, the SKN-1::GFP fluorescence intensity was measured in the gut area below the pharynx. SOD-3 fused with GFP was assessed in the whole CF1553 worm. For TJ375, the anterior area of the pharyngeal bulb was used in the quantification of HSP-16.2::GFP expression. In the same way as for SOD-3, the GST-4 expression in the transgenic strain CL2166 was also obtained from the entire worm.

### 2.10. Target Genes Silencing by RNAi Technology

The inhibition of target gene expression was performed using the RNAi technique. This experiment was applied to the paralysis test and to the transgenic reporter strains. *E*. *coli* HT115 expressing *DAF-16/FOXO* (accession number: AF032112.1), *SOD-2* (accession number: NM_059889.7), *SOD-3* (accession number: NM_078363.9) (Cultek SL, Madrid, Spain), *SKN-1/NRF2* (accession number: NM_171347.7) and *HSP-16.2* (accession number: NM_001392482.1) (Sources BioScience, Nottingham, UK). RNAi plates were prepared by adding dsRNA to NGM containing 25 μg/mL carbenicillin and 1 mM Isopropyl β-D-1-thiogalactopyranoside (IPTG). F0 (L3-L4 synchronized worms) growing in non-treated plates were moved to the RNAi dishes. When animals reached fertile age, the eggs (F1) were isolated through the bleaching method. For CL4176, used in the paralysis test, the eggs were placed into plates containing HOFE 100 μg/mL treatment with each RNAi and a plate without the RNAi. Next, the paralysis experiment was carried out as explained above. For TJ356, LD1, TJ375 and CF1553 GFP-reporter strains, eggs were placed on plates with or without the respective RNAi. The standard protocol explained above was followed from this step to verify the effectiveness of the RNAi gene inhibition.

### 2.11. Statistical Analysis

Prior to analyzing variables for differences between groups, normality and homogeneity of variance were analyzed by Kolmogorov-Smirnov and Levene tests, respectively. Variables following normality were evaluated through Student *t*-test. Variables not following normal distribution were assayed via Mann-Whitney-U and Kruskal-Wallis tests. Statistical significance was considered for *p* < 0.05. Statistical analysis for longevity was performed applying the Log-Rank test. SPSS 24.0 (IBM, Armonk, NY, USA) was used in all cases.

## 3. Results

### 3.1. Total Phenol and Flavonoid Content, and Total Antioxidant Capacity

Flavonoid and phenolic contents, as well as global antioxidant capacity, measured by means of three different assays (DPPH, FRAP and ABTS) are collected in Table 1.

### 3.2. Hydroxytyrosol Identification via UPLC-QTOF-MS/MS

**Figure 1:**
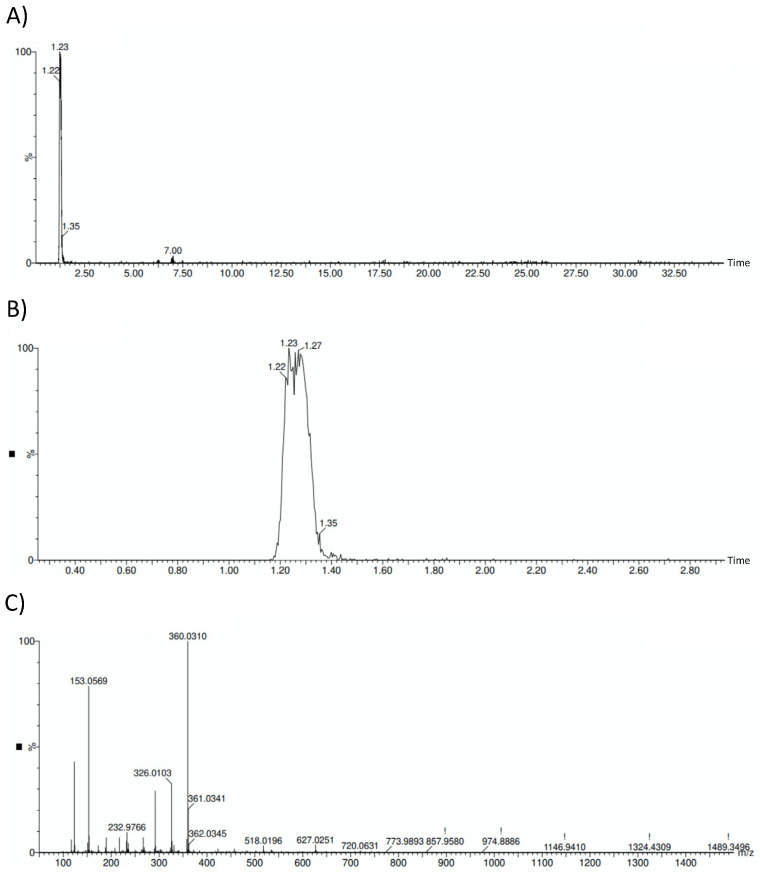
HOFE chromatograms and mass spectrum. (**A**) General HOFE chromatogram in which hydroxytyrosol peak appears at a retention time of 1.23 min. (**B**) Amplified chromatogram with detail of hydroxytyrosol peak. (**C**) Negative mass spectrum of HOFE. Hydroxytyrosol has a negative mass of 153.0569.

Verification of hydroxytyrosol presence on the sample is reflected in Figure 1, where the HOFE chromatograms and mass spectrum are gathered.

### 3.3. In Vivo Toxicity Assessment

**Figure 2:**
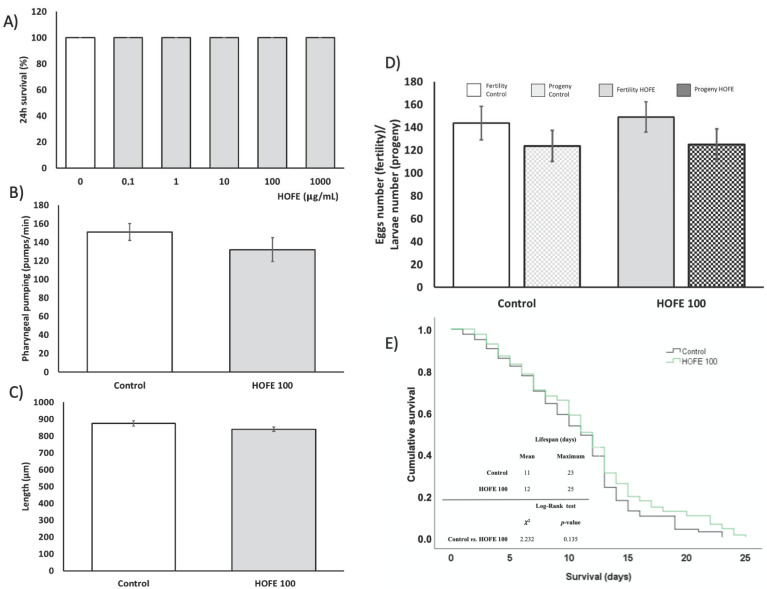
Toxicity assessment of HOFE in N2 *C. elegans* strain. (**A**) Lethality. (**B**) Pharyngeal pumping. (**C**) Growth. (**D**) Reproduction and fertility. (**E**) Kaplan-Meier survival curves representative of long-term in vivo toxicity. Results are expressed as mean ± SEM.

Before the assessment of the treatment effect on AD features, the toxicity of HOFE, both at the short and long term, was evaluated in the *Caenorhabditis elegans* model. Figure 2A shows that 24 h dose-response lethality manifested a non-toxic effect of the treatment at any of the assessed concentrations (0, 0.1, 1, 10, 100 and 1000 μg/mL), with 100% survival after 24 h of exposure for every dosage. The toxic impact on worm metabolism and development were assayed by the pharyngeal pumping and growth tests, respectively. Pharyngeal pumping determines the food intake of the organisms, and a delayed growth can be indicative of dysfunction of several developmental processes. In this regard, no differences were found between 100 μg/mL- treated worms and the control group in these parameters (Figure 2B,C). Likewise, egg laying ability and egg viability were not affected by HOFE at the tested dose, as reflected in Figure 2D. On the other hand, the potential long-term toxicity of HOFE was evaluated by the survival curve, also in the N2 wild strain. The treatment did not modify worm longevity according to Log-Rank test (*p* = 0.135), exhibiting similar mean and maximum lifespan as the control group (Figure 2E).

### 3.4. Effect of HOFE against AAPH-Induced Oxidative Stress

**Figure 3:**
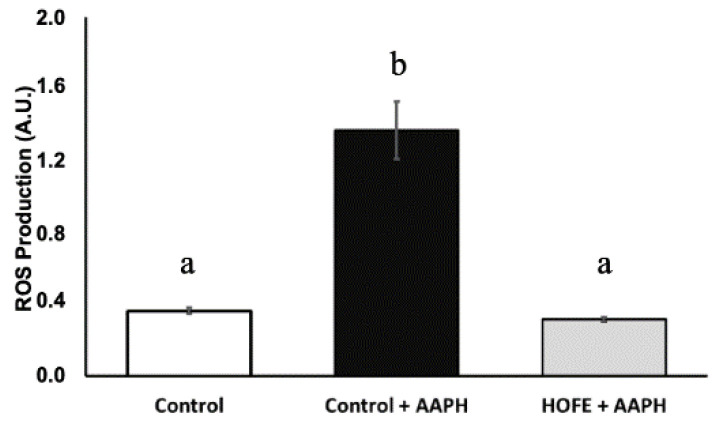
Effect of the hydroxytyrosol-rich olive fruit extract HOFE at 100 μg/mL against AAPH-induced oxidative stress. Different superscript letters over results columns mean statistically significant differences (*p* < 0.05). Results are expressed as mean ± SEM. AAPH: 2,2′-Azobis(2-methylpropionamidine) dihydrochloride.

Oxidative stress was induced by the stressor AAPH, and DCFDA dye was used for the measurement of ROS content (Figure 3). AAPH induction led to a statistically significantly higher ROS content in live worms compared to baseline control animals. On the other hand, treatment with 100 μg/mL HOFE was able to protect the worms from the oxidizing agent, as shown by the lower ROS content.

### 3.5. Effect of HOFE against Aβ-Induced Paralysis

**Figure 4:**
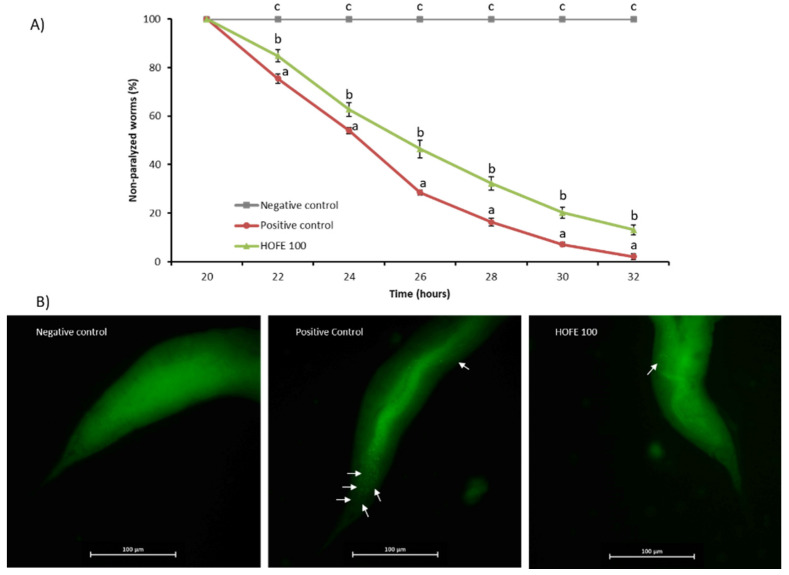
HOFE (100 μg/mL) effect on Aβ-induced paralysis CL4176 nematodes. (**A**) Paralysis curve. For each time, different letters represent statistically significant differences (*p* < 0.05) between groups. Results are expressed as mean ± SEM. (**B**) Typical images from each experimental group presenting Thioflavin T aggregates in worms at 26 h after temperature shifting (40× magnification). Aβ aggregates are indicated with white arrows. Negative control is represented by the non-paralyzable strain CL802. Positive control is the non-treated CL4176.

A paralysis test was performed to analyze any potential effectiveness of the extract on Aβ-induced toxicity. For this purpose, CL4176 strain, which expresses the human Aβ1-42 peptide in muscle cells in a temperature inducible way and leads to a paralysis phenotype, was used. The percentage of non-paralyzed worms depending on number of hours at a non-permissive temperature (25 °C) is represented in Figure 4A. Results showed a significant delay of the paralysis in the 100 μg/mL HOFE treated group from 22 h after temperature upshift until the end of the registered experimental time. These findings suggest the protection of the treatment against Aβ-induced toxicity correlated with the amount of Aβ deposits found in muscle cells, as revealed by Thioflavin T staining (Figure 4B). In fact, non-treated CL4176 worms (positive control) showed a large content of Aβ aggregates. In contrast, HOFE treated worms exhibited less Thioflavin T-positive aggregates.

**Figure 5:**
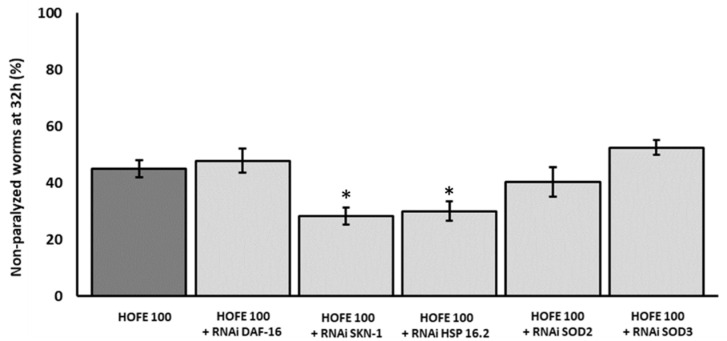
Effect of the different RNAi (*HSP-16.2*, *SOD-3*, *SOD-2*, *SKN-1*, and *DAF-16*) in a paralysis assay in CL4176 worms treated on HOFE at 100 μg/mL. Data are presented as mean ± standard error of the mean. An * means significant differences (*p* < 0.05) with respect to worms non-exposed to RNAi and treated on HOFE.

After observations on Aβ aggregation, RNAi technology was applied to try to decipher molecular mechanisms under the positive effects exerted by HOFE. Thus, a new paralysis experiment was performed by using CL4176 worms treated with HOFE and exposed to RNAi of the different genes of interest. Figure 5 displays the non-paralyzed nematodes (%) after 32 h of temperature shifting in the transgenic model knocked-down for the genes *SKN-1/NRF2*, *DAF-16/FOXO*, *SOD-3*, *SOD-2* and *HSP-16.2*. *HSP-16:2* and *SKN-1/NRF2* RNAi reduced (*p* < 0.05) non-paralyzed nematodes (%) compared to the HOFE treated worms without RNAi as control. This means that SKN-1/NRF2 and DAF-16/FOXO should be involved, at least in part, in the observed HOFE protective effect. SOD-3, SOD-2 and DAF-16/FOXO, did not modify the degree of paralysis and they were not involved, at least under the present experimental conditions, in the protection conferred by the HOFE treatment against Aβ aggregation.

### 3.6. Effect of HOFE against Tau Proteotoxicity

**Figure 6:**
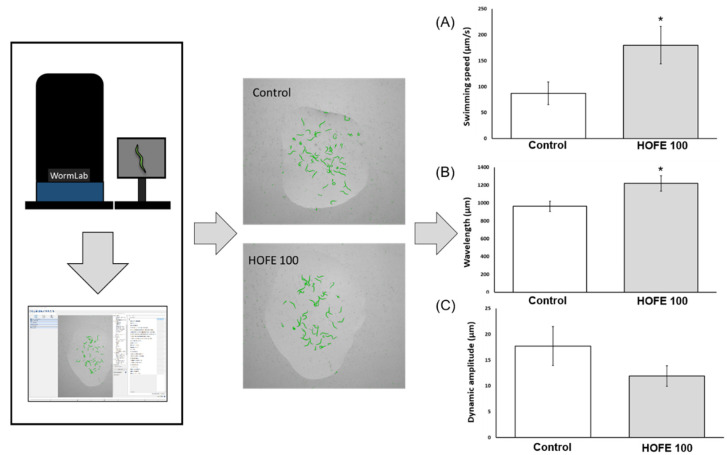
Effects of HOFE on Tau-induced altered locomotive behavioral phenotype in BR5706 transgenic strain. (**A**) Swimming speed. (**B**) Wavelength. (**C**) Dynamic amplitude/Stretching effort. Results are expressed as mean ± SEM. An * means significant differences (*p* < 0.05) with respect to control.

The presence of aggregates of the tau protein is a feature of AD, together with Aβ deposits, oxidative stress, and others. Tau aggregation was assessed in vivo by using the BR5706 strain. This transgenic model expresses the pro-aggregate human Tau protein in a constitutive and pan-neuronal way, leading to locomotion defects, mainly in adulthood. WormLab station and software were used for analyzing swimming locomotive behavior. As shown in Figure 6, treatment with HOFE 100 μg/mL was able to improve swimming speed as well as body waviness. However, stretching effort was not modified by the treatment in BR5706 worms.

### 3.7. Effect of HOFE in the DAF-16::GFP, SKN-1::GFP, SOD-3::GFP, HSP16.2::GFP and GST-4::GFP Expression

**Figure 7:**
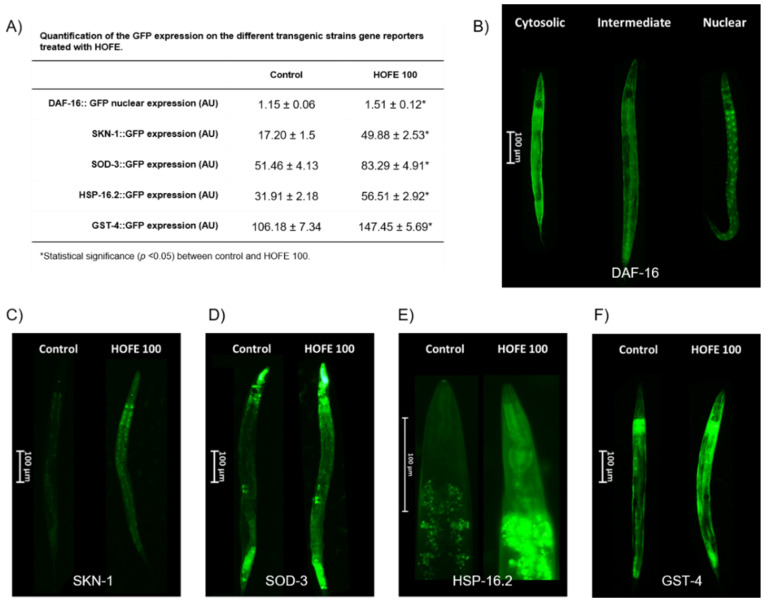
Effects of the 100 μg/mL HOFE on transgenic worms. (**A**) GFP quantification. (**B**) Representative pictures of DAF-16::GFP categorization. (**C**) Representative pictures of SKN-1::GFP worms. (**D**) Representative pictures of SOD-3::GFP nematodes. (**E**) Representative pictures of HSP-16.2::GFP worms. (**F**) Representative pictures of GST-4::GFP nematodes. Results are presented as the mean ± standard error of the mean. * Significant difference (*p* < 0.05) vs. control.

Effects of the 100 μg/mL HOFE on GFP-reporter transgenic strains are presented in Figure 7. TJ356 worms, both control group and treated with HOFE 100 μg/mL, were categorized as nuclear, intermediate or cytosolic in accord with the presence of DAF-16/FOXO::GFP nucleation (Figure 7B). HOFE treatment-induced nucleation of DAF-16/FOXO (Table shown in panel A of Figure 7 and Figure 7C). Regarding SKN-1 transcription factor, treatment also increased nuclear translocation in the transgenic strain LD1 (Table shown in panel A of Figure 7C). For SOD-3, HSP16.2 and GST-4 transgenic strains, fluorescence intensity was higher (*p* < 0.05) in the treated groups vs. the control (Table shown in panel A of Figure 7). Representative images for the three strains highlight the expression along the entire worm length, but mainly in head and tail, for SOD-3 (Figure 7D), in the anterior pharyngeal bulb for HSP-16.2 (Figure 7E) and in the whole nematode for GST-4 (Figure 7F).

**Figure 8:**
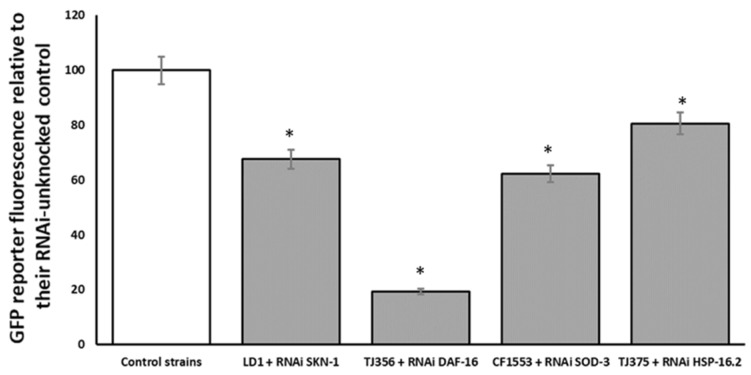
Effect of *SKN-1*, *DAF-16:2*, *SOD-3* and *HSP16.2* RNAi on respective GFP transgenic strains for the same respective genes. Results are presented as % vs. control. An * indicates significant differences (*p* < 0.05) vs. control.

Additionally, strains endowed with the GFP reporter were matched against RNAi for its respective transgene to confirm the inhibitory effect of the RNAi on the selected gene. This assay was conducted to confirm that the results obtained in the paralysis assay were attributed to capacity of the specific RNAi technology silencing gene. As shown in Figure 8, all assayed strains were affected by their respective RNAi.

## 4. Discussion

This study was designed to evaluate an olive-derived extract enriched in hydroxytyrosol on different features of AD. Concerning the characterization of the extract, mass spectrometry analysis confirmed that the extract was rich in hydroxytyrosol (20%). Concerning total phenolic and flavonoid contents, the studied extract showed higher values for both parameters in comparison with different methanolic and ethanolic olive fruit extracts from Tunisia [23,24] and China [25]. In the same way, in vitro antioxidant activity of the studied extract was higher for ABTS and DPPH in comparison with different methanolic and ethanolic olive fruit extracts from Tunisia [23,24]. Martinez et al. also evaluated the antioxidant activity and phenolic content of an olive fruit extract enriched in hydroxytyrosol (11.25%) from Spain [26]. In this context, the HOFE assayed in the present research showed higher values of total phenolic content, as well as FRAP activity, in comparison to the extract analyzed by Martinez et al. These differences could be attributed to the different proportion of hydroxytyrosol in the two extracts. Furthermore, variations in geographic location and climatology, which have been reported to exert important changes in the phenolic and antioxidant profile, even in olive fruits for the same country, might account for such differences [23,24]. In the same way, stage of ripening as well as position of the olive fruit on the tree (low or upper position) has been related to variations in the phenolic and flavonoid composition [27].

Prior to investigating putative usefulness of the HOFE extract regarding AD features, toxicity assessment was conducted. Firstly, acute toxicity tests showed non-toxic effects of the HOFE, as indicated by the 24 h lethality test. This lack of toxicity was also found for metabolic activity, growth, fertility, and reproduction assays. Similar results were obtained with other olive tree by-products, such as olive leaf extract, which did not affect, or even increase, clearance of food (used to test metabolic status) [28]. On the contrary, Luo et al. [28] reported a study showing that olive leaf extract was able to increase egg laying in treated worms. In the present research, an additional experiment was developed to test long-term toxicity. Specifically, lifespan was not affected by HOFE treatment. In contrast, it has been shown that 250 μg/mL of olive fruit extract enriched in hydroxytyrosol (6%) or 250 μg/mL of pure hydroxytyrosol increased lifespan in *C. elegans* [29,30]. To summarize toxicity assays, hydroxytyrosol-rich extract used in the present research was not reported to be toxic.

Among the most early and critical events studied in the pathophysiology of AD, oxidative stress is a conserved feature presented in both nematodes and humans [31,32]. Therefore, the capacity of HOFE to enhance resistance against oxidative stress was evaluated. HOFE prevented ROS rising after oxidant exposition in N2 nematodes. In the same way, other olive tree by-products, such as olive leaf, have demonstrated the capacity to reduce ROS content in vitro [33] as well as in *C. elegans* [28]. Some natural olive tree by-products, as well as several polyphenols present in the olive fruit, have been evaluated due to direct antioxidant activities in vitro [34]. In the present study, HOFE supplementation has been revealed to promote nucleation of DAF-16/FOXO::GFP and to increase gene expression of SKN-1/NRF2::GFP. Similar results have been reported for DAF-16 for an olive leaf extract that was related to the induction of antioxidant enzymes in *C. elegans* [28]. DAF-16/FOXO and SKN-1/NRF2 are two important transcription factors involved in numerous processes which have been linked to AD progression as well as xenobiotic and oxidative stress responses. Among the antioxidant enzymes coded by DAF-16/FOXO and SKN-1/NRF2, the expression of SOD-3 and GST-4 were evaluated. SOD-3 encodes MnSOD mitochondrial isoforms which act to convert superoxide into molecular oxygen and hydrogen peroxide, while GST-4 regulates phase II and ROS detoxification. MnSOD mitochondrial isoforms’ overexpression has been related to a reduction in Aβ plaque deposition [35] whereas a GST-4 up-regulation has been linked to a reduction in Aβ toxicity [36]. In the present research, HOFE exposition increased the GFP expression in both of the mentioned antioxidant enzymes. In the same way, olive leaf has been shown to be effective in increasing antioxidant activity of SOD as well as of GSH-Px in *C. elegans* [28]. Beyond olive tree by-products, some compounds present in the olive fruit, such as oleuropein, have been shown to increase gene expression of *SOD-3* [37], whereas tyrosol has demonstrated its ability to increase GST-4 [38,39,40] but not SOD-3 [38,40] expression in *C. elegans.*

Health benefits of olive oil intake are widely known. Among observed protective effects, olive tree by-product consumption has been shown to reduce AD-related symptoms in animals and in clinical trials [41,42]. These positive effects have been associated with the good content of olive by-products of several bioactive compounds, like oleuropein and hydroxytyrosol. To our knowledge, this is the first research that uses olive fruit as a therapeutic agent against AD-related features in *C. elegans*. The deposition of Aβ peptide is one of the two main histopathological hallmark lesions presented in AD [43]. Amyloid cascade hypothesis has been proposed as a central key of AD pathology. Notwithstanding this, some results indicate that the hyperphosphorylation of tau protein, another AD histopathological hallmark lesion, is necessary for amyloid-related toxicity [44]. Here, both pathological mechanisms were assayed to deepen the potential therapeutic effect of HOFE in both AD features (beta amyloid and tau related aspects). CL4176 transgenic worms express human Aβ in muscle cells and were used to evaluate HOFE against Aβ-induced toxicity. According to the results, HOFE delayed the paralysis phenotype related to Aβ-induced toxicity. The Thioflavin-T method uncovered the fact that the lower Aβ toxicity was caused by lower Aβ aggregation in HOFE-treated worms. Several phenolic compounds present in olive fruit have demonstrated potential anti-Aβ effects in vivo and in vitro. To be more specific, hydroxytyrosol, tyrosol and oleuropein have been shown to be effective in the reduction of Aβ-induced toxicity in vitro [45,46,47,48,49,50]. Additionally, hydroxytyrosol has been shown to restore impaired cognitive functions in different AD mice models [48,51,52]. RNAi technology was then used to investigate HOFE mechanisms of action. Only *SKN-1/NRF2* knockout worms showed a higher degree of paralysis than nematodes not exposed to RNAi. These results suggest the involvement of *SKN-1/NRF2* in observed HOFE effects against Aβ-induced toxicity. SKN-1/NRF2 is involved in the expression of antioxidant enzymes and other protective effects. In fact, NRF2 activation has been shown to decrease Aβ production through the reduction of BACE1 transcription levels. In addition, NRF2 may also reduce Aβ aggregation levels and decrease the Aβ toxicity [53]. Noteworthy in the present study is the fact that SOD-3 expression was enhanced, as shown by the study of the GFP strain for this mitochondrial antioxidant enzyme, although no effects were directly observed regarding applied RNAi for the *SOD-3* gene in the paralysis assay. On the other hand, HSP-16.2 was involved in the protective effect of the extract against Aβ-induced toxicity when RNAi technology was used. According to the literature, HSP-16.2 has been shown to interfere directly with Aβ oligomerization pathways which leads to reduction of Aβ toxic species formation in *C. elegans* [54,55]. Therefore, these results suggest that the observed anti-Aβ effects of HOFE might be mediated via *SKN-1/NRF2* and *HSP-16.2*. However, even though RNAi studies in strain CL4176 show a direct effect of *SKN-1* and *HSP -16:2*, the fact that GFP-reporter assays found overexpression of SKN-1 and HSP- 16:2, but also of DAF-16, SOD-3, and GST-4, could indicate that these last transcription factor and antioxidant enzymes could have some kind of implication in the protection of HOFE, reducing toxicity in general and lowering environmental stressors. These effects could be mediated through hormesis phenomena or others that need to be studied in the future. Finally, the verification of the effects of the different RNAi clones in the respective transgenic strains with GFP-reporter presented in Figure 8 shows different degrees of expression depending on the RNAi and the strain with GFP-reporter. However, these differences should not be interpreted as greater or lower efficiency of the respective RNAi since it has been reported that some strains are highly sensitive to RNAi, whereas other strains vary greatly in their RNAi responses [56,57,58,59]. Thus, the objective of this assay was to see if there were differences with respect to the control, but not between different RNAi strains and clones.

On the other hand, the effect of HOFE against tau-induced neurotoxicity was also evaluated in *C. elegans*. In this regard, BR5706 strain was used to analyze locomotion defects related to neuronal toxic pro-aggregating accumulation of human tau protein. According to the results obtained in the present research, HOFE treatment improved the locomotive alterations related to toxic accumulation of tau protein in neurons. In relation to that, similar results were obtained with an olive leaf extract enriched in oleuropein (40%) against tau neurotoxicity in *C. elegans*. In this context, the extract (100 µg/mL) reduced the locomotive alterations related to tau toxicity on day one of adulthood in the BR5706 strain. In this research, authors showed that SKN-1/NRF2 and DAF-16/FOXO signaling, as well as HSP-16.2 overexpression, were involved in the protective effect observed [60]. In the same way, the effectiveness of an hydroxytyrosol enriched (6%) olive pulp extract was also evaluated in a *C. elegans* model of Parkinson. In this context, the extract did not affect neuronal α-synuclein accumulation or locomotive behavior when assayed at 250µg/mL in the first three and seven days of adulthood. However, in the mentioned study, the extract was able to reduce the accumulation of α-synuclein after 12 days of exposure [29]. In the present research, the locomotive test was performed on day one of adulthood with promising effects of HOFE treatment at the dosage used. However, the long-term, as well as higher, dosage of HOFE in the constitutive pro-aggregating tau protein strain have not been explored yet and could open an interesting research field.

## 5. Conclusions

**Figure 9:**
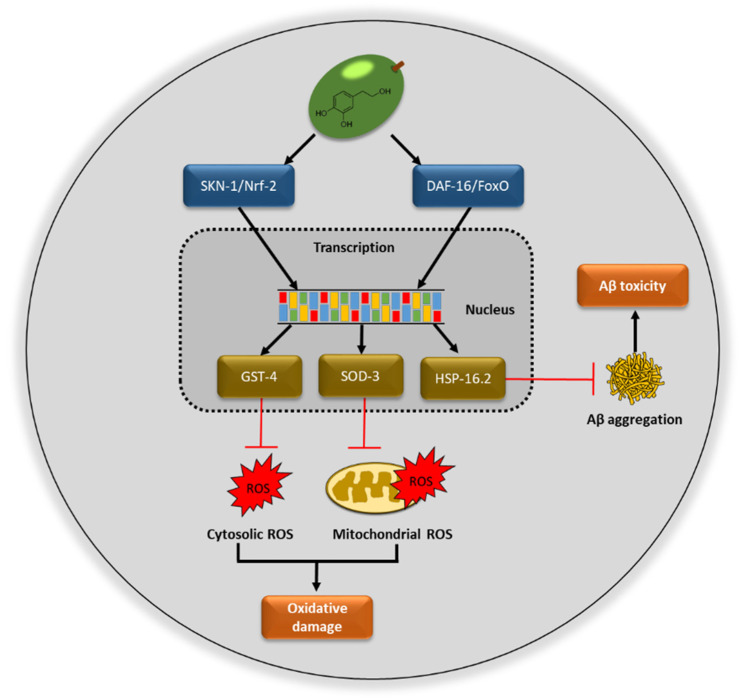
Possible mechanisms of action of hydroxytyrosol rich olive fruit extract (HOFE) against AD features investigated in the model *Caenorhabditis elegans*. ROS, reactive oxygen species.

The extract rich in hydroxytyrosol coming from olive fruits (HOFE) studied in the present research offered good antioxidant properties. According to the in vivo experiments performed, HOFE did not exert short- or long-term toxicity in *Caenorhabditis elegans*. Moreover, this research has found, as its main results, the positive effects of HOFE on some features of Alzheimer disease also investigated in the nematode model *C. elegans*, namely beta amyloid aggregation, tau proteotoxicity and oxidative stress. Figure 9 summarizes possible mechanisms of action of HOFE. Together, these results feature interest in the olive fruit and HOFE, particularly if it is considered that the extract assayed in the present research has been authorized for human supplementation, which grants the design and development of tests in higher animal models of AD and human trials.

## Figures and Tables

**Table 1:** Total phenolics content, total flavonoids content and total antioxidant capacity of the Olea Europaea fruit extract 20% rich in hydroxytyrosol.

Parameter	Mean ± SEM
Total flavonoids content (mg catechin equivalent/g DE)	377.9 ± 18.4
Total phenolic content (mg gallic acid equivalent/g DE)	202.4 ± 12.8
DPPH (mM TE/g DE)	3.32 ± 0.28
FRAP (mM TE/g DE	3.16 ± 0.08
ABTS (mM TE/g DE)	1.47 ± 0.09

Abbreviations: ABTS: 2,2′-azinobis(3-ethylbenzothiazoline-6-sulfonic acid); DE: dry extract; DPPH: 2,2-diphenyl-1-picryl-hydrazyl-hydrate; FRAP: ferric reducing antioxidant power; TE: trolox equivalent.

## Data Availability

The data presented in this study are available on request from the corresponding author. The data are not publicly available yet because funded grants are still ongoing.
